# Supplementary material for: Learning curve and surgical outcome of robotic assisted colorectal surgery with ERAS program
Source: Sci Rep. 2022 Nov 29;12:20566. doi: 10.1038/s41598-022-24665-w (PMC9709162; doi:10.1038/s41598-022-24665-w)
Supplement: Supplementary file 1 — Supplementary Information 1. [file 41598_2022_24665_MOESM1_ESM.zip › LSC Raw data-Ñ[▒K/112/P_ñw╝╨░O▒Kñσ.pdf]

[redacted]

Pathologic diagnosis:
1. Sigmoid colon, laparoscopic anterior resection ---
a. Adenocarcinoma, moderately differentiated, arising in a tubular adenoma. Tumor invades to mesocolic soft tissue.
b. Tubular adenomas.
The bilateral cut ends and mesocolic lymph nodes (0/18) are free of the above lesions.
2. IMA root, dissection --- Unremarkable lymph nodes (0/1).
3. Cut end, distal, resection --- Negative for malignancy.

Ancillary study for diagnosis:
1. Re-cut done for section F.
2. IHC stains for MLH1, MSH2, PMS2 and MSH6 are done on section L. All above markers are preserved.

Prognostic and predictive factor:
1. Tumor size: 2x1.8 cm.
2. Depth of invasion: Mesocolic soft tissue.
3. Histologic grade: G2: Moderately differentiated.
4. Angiolymphatic invasion: Not identified.
5. Perineural invasion: Not identified.
6. Tumor deposits (discontinuous extramural extension): Not identified.
7. Macroscopic tumor perforation: Not identified.
8. Surgical Margins:
a) Proximal Margin: Uninvolved by invasive carcinoma, distance of tumor from margin: 135 mm.
b) Distal Margin: Uninvolved by invasive carcinoma, distance of tumor from margin: 30 mm.
c) Radial or Mesenteric Margin: Uninvolved by invasive carcinoma, distance of tumor from margin: more than 10 mm.
9. Lymph node status: Uninvolved (0/19).
10. Extranodal involvement: Not identified.
11. Tumor regression grading S/P CCRT: No known presurgical therapy.
12. Type of polyp in which invasive carcinoma arose: Tubular adenoma.
13. Additional pathologic findings: Adenoma(s).
14. Pathological TNM Stage: pT3N0 (According to the eighth edition, American Joint Committee on Cancer Staging Guidelines for Tumors).
15. TNM descriptors: Not applicable.

Gross description:
The specimen consists of 1) a segment of colon, 18.3 cm in length and 5.5 cm in proximal cut end circumference, 5 cm in distal circumference, and mesocolic soft tissue. A gray tan firm annular elevated and centrally ulcerative lesion, 2x1.8 cm, is noted, 13.5 cm and 3 cm respectively to the proximal and distal cut ends. On cross section, the lesion involves underlying muscularis propria with more than 10 mm to the radial margin. There are also 4 sessile polyps on the mucosa. The first polyp is 0.6x0.5x0.2 cm in size and 15 cm to the distal cut end; the second one is 0.6x0.5x0.2 cm in size and 14 cm to the distal cut end; the third polyp is 1x0.2x0.1 cm in size and 12 cm to the distal cut end; and the last one is 1x0.4x0.3 cm in size and 1.7 cm to the distal cut end. A blackish polypoid lesion, 0.6x0.5x0.5 cm in size and 3 cm away from the distal cut end, is noted. The both cut ends are free of the above lesions grossly. The serosa is inked by blue dye, and the radial margin is inked by yellow dye. 2) IMA root, a piece of tan yellow soft tissue, 2.5x2x0.6 cm. 3) distal cut end, a piece of tan mucosa-covered soft tissue, 3.1x2x1 cm. All for section: A) specimen 2; Representative part for section: B) specimen 3 C) proximal cut end D) the 1st polyp E) the 2nd polyp F) the 3rd polyp G) the blackish polypoid lesion H) the 4th polyp J) radial margin K-L) lesion M-N) lymph nodes.

Note:
1. The interpretation of MMR panel and recommended studies are as below: (1) No loss of nuclear expression of MMR proteins: low probability of microsatellite instability-high (MSI-H) . (2) Loss of nuclear expression of MLH1 and PMS2; testing for methylation of the

nuclear expression of MLH1 and PMS2: testing for methylation of the MLH1 promoter and/or mutation of BRAF is indicated (the presence of a BRAF V600E mutation and/or MLH1 methylation suggests that the tumor is sporadic and germline evaluation is probably not indicated; absence of both MLH1 methylation and of BRAF V600E mutation suggests the possibility of Lynch syndrome, and sequencing and/or large deletion/duplication testing of germline MLH1 may be indicated). (3) Loss of nuclear expression of MSH2 and MSH6: high probability of Lynch syndrome (sequencing and/or large deletion/duplication testing of germline MSH2 may be indicated, and, if negative, sequencing and/or large deletion/duplication testing of germline MSH6 may be indicated). (4) Loss of nuclear expression of MSH6 only: high probability of Lynch syndrome (sequencing and/or large deletion/duplication testing of germline MSH6 may be indicated). (5) Loss of nuclear expression of PMS2 only: high probability of Lynch syndrome (sequencing and/or large deletion/duplication testing of germline PMS2 may be indicated). (6) Please note: There are exceptions to the above IHC interpretations. These results should not be considered in isolation, and clinical correlation with genetic counseling is recommended to assess the need for germline testing.
2. This case has been peer reviewed by two doctors.
#T-59470_2 #M-82633_2 2 1105 S0000F
#T-C4560_2 #M-09413_2 0 1105 000000

已電子簽章

敬告：本單僅供醫師參考使用，不具任何申請效力！！
請病患妥為保管，若不慎遺失需自負個人資料外洩責任！
